# Impact of audit and feedback with action implementation toolbox on improving ICU pain management: cluster-randomised controlled trial

**DOI:** 10.1136/bmjqs-2019-009588

**Published:** 2019-07-01

**Authors:** Marie-José Roos-Blom, Wouter T Gude, Evert de Jonge, Jan Jaap Spijkstra, Sabine N van der Veer, Niels Peek, Dave A Dongelmans, Nicolette F de Keizer

**Affiliations:** 1 Department of Medical Informatics, Amsterdam Public Health Research Institute, Amsterdam UMC, University of Amsterdam, Amsterdam, The Netherlands; 2 National Intensive Care Evaluation (NICE) Foundation, Amsterdam, The Netherlands; 3 Department of Intensive Care Medicine, Leiden University Medical Center, Leiden, The Netherlands; 4 Department of Intensive Care Medicine, Amsterdam UMC, Vrije Universiteit Amsterdam, Amsterdam, The Netherlands; 5 Centre for Health Informatics, Division of Informatics, Imaging and Data Sciences, Faculty of Biology, Medicine and Health, Manchester Academic Health Science Centre, University of Manchester, Manchester, UK; 6 NIHR Greater Manchester Patient Safety Translational Research Centre, Manchester Academic Health Science Centre, University of Manchester, Manchester, UK; 7 Department of Intensive Care Medicine, Amsterdam UMC, University of Amsterdam, Amsterdam, The Netherlands

**Keywords:** pain, feedback, intensive care units, quality improvement, dashboard, action implementation toolbox

## Abstract

**Background:**

Audit and feedback (A&F) enjoys widespread use, but often achieves only marginal improvements in care. Providing recipients of A&F with suggested actions to overcome barriers (action implementation toolbox) may increase effectiveness.

**Objective:**

To assess the impact of adding an action implementation toolbox to an electronic A&F intervention targeting quality of pain management in intensive care units (ICUs).

**Trial design:**

Two-armed cluster-randomised controlled trial. Randomisation was computer generated, with allocation concealment by a researcher, unaffiliated with the study. Investigators were not blinded to the group assignment of an ICU.

**Participants:**

Twenty-one Dutch ICUs and patients eligible for pain measurement.

**Interventions:**

Feedback-only versus feedback with action implementation toolbox.

**Outcome:**

Proportion of patient-shift observations where pain management was adequate; composed by two process (measuring pain at least once per patient in each shift; re-measuring unacceptable pain scores within 1 hour) and two outcome indicators (acceptable pain scores; unacceptable pain scores normalised within 1 hour).

**Results:**

21 ICUs (*feedback-only* n=11; *feedback-with-toolbox* n=10) with a total of 253 530 patient-shift observations were analysed. We found absolute improvement on adequate pain management in the *feedback-with-toolbox* group (14.8%; 95% CI 14.0% to 15.5%) and the *feedback-only* group (4.8%; 95% CI 4.2% to 5.5%). Improvement was limited to the two process indicators. The *feedback-with-toolbox* group achieved larger effects than the *feedback-only* group both on the composite adequate pain management (p<0.05) and on measuring pain each shift (p<0.001). No important adverse effects have occurred.

**Conclusion:**

Feedback with toolbox improved the number of shifts where patients received adequate pain management compared with feedback alone, but only in process and not outcome indicators.

**Trial registration number:**

NCT02922101.

## Introduction

Patients admitted to intensive care units (ICUs) receive complex and high-tech treatment which make them high healthcare cost consumers and susceptible to harm. Therefore, ICUs continuously strive to improve the quality of care they deliver.^1^ One area of focus concerns management of pain. More than 30% of the ICU patients experience moderate to severe pain at rest, and this increases to 50% during common care procedures such as chest tube and wound drain removal, and arterial line insertion.^2 3^ Pain is associated with discomfort, sleep deprivation, and an increased morbidity, mortality and length of stay.^4–6^ To assess pain, validated tools exist such as the Visual Analogue Scale (VAS) or Numeric Rating Scale (NRS) in patients able to self-report,^7^ and the Critical-Care Pain Observation Tool (CPOT) or Behavioral Pain Scale (BPS) in patients not able to self-report.^8 9^ The severity of pain is often underestimated by ICU professionals, and as a consequence pain is treated inadequately.^10 11^ Providing ICUs with performance data about their pain management practice such as audit and feedback (A&F) can be an effective strategy to improve the quality of ICU pain management.^12^

A&F is commonly used for improving the quality of care in the ICU.^12–15^ A&F provides a summary of clinical performance over a specified period of time,^16^ and is an effective strategy to improve quality of care, but its effects are variable and often marginal.^17 18^ When baseline performance is low and the feedback is provided repeatedly, by a supervisor or senior colleague in verbal and written formats, and includes specific targets and action plans on how to change behaviour, A&F may be more effective.^17 19^ Information on how these factors should be operationalised in order to deliver effective A&F interventions is still unclear.^20^ Head-to-head comparisons of interventions with different approaches are necessary to increase the understanding and effect size of A&F.^21^

Control Theory^22^ predicts that health professionals confronted with feedback indicating a discrepancy between current and desirable practice will be prompted to take action to improve practice, until the discrepancy is eliminated. In practice, however, health professionals often lack the skills, time, capacity or knowledge to interpret feedback and formulate what improvement action is necessary.^17 23–25^ Similarly, they may not be aware of barriers that could hamper the implementation of their intended actions.^26^ We hypothesised that augmenting A&F with an action implementation toolbox, that is, a list of potential barriers in the care process and suggested actions with supporting materials to facilitate the planning and implementation of actions, would help physicians to turn their intention into action and would increase the likelihood that actions will be completed.^27^

We aimed to assess the effect of adding an action implementation toolbox to an electronic A&F intervention. Our application area is quality of pain management in ICUs. We hypothesised that ICUs in both study groups would improve on pain management, but that ICUs who had access to the toolbox would achieve larger improvements than those who had not.

## Methods

### Study design

This study was a pragmatic two-armed cluster randomised controlled trial using block randomisation, with randomly permuted blocks of two or four, each consisting of an equal number of ICUs in the *feedback-only* group and *feedback-with-toolbox* group. Cluster randomisation was chosen because the intervention is implemented at the level of ICUs rather than individual professionals. Randomisation of individual professionals or patients was not feasible because contamination would have occurred as strategies for improvement would have been shared between professionals within the same practice.^28^ A researcher, unaffiliated with the study and blinded to the identity of the units, performed the randomisation. Investigators were, due to the nature of the intervention, not blinded to the group allocation of an ICU. ICUs in both groups received electronic feedback on new pain management indicators. Both groups also received an action planning template, but ICUs in the *feedback-with-toolbox* group additionally received an integrated list of potential barriers and suggested actions with supporting material to overcome these barriers. ICUs were aware that there were two variations of the intervention being evaluated, but they were not told what aspect (ie, the toolbox) was randomised (see description of intervention). More details can be found in the study protocol.^27^ The study results are reported according to the CONSORT statement for cluster-randomised trials.^29^

### Setting

In the Netherlands, there are 120 hospitals of which 82 have an adult ICU. Currently, all these 82 ICUs collect data on demographics, physiology and clinical diagnoses of all patients admitted to their ICU and deliver these data monthly to the Dutch National Intensive Care Evaluation (NICE) registry.^30^ Collected data are checked by registry staff for internal consistency, by performing onsite data quality audits and training local data collectors.^31^ The NICE registry has enabled participating ICUs to quantify and improve their quality of care by offering them feedback with benchmark information on patient outcomes such as mortality and length of stay for more than 20 years. Each participating ICU receives biannual reports and has access to an online tool that enables them to perform additional analyses on their data at any time.

In 2016, the NICE foundation introduced four new quality indicators to assess and improve the ICUs’ performance regarding pain management. The development of the indicators followed a modified RAND procedure and was performed in collaboration with pain management experts from the field.^32^ The resulting indicator set reflects adequate pain management for each ICU patient during each 8-hour shift, meaning that the unit of observation is one patient-shift, composed by two process and two outcome indicators (see online supplementary table 1 for details):

10.1136/bmjqs-2019-009588.supp1 Supplementary data

1. Proportion of patient-shifts during which pain was measured at least once (process);
2. Proportion of patient-shifts during which an unacceptable pain score (defined as VAS/NRS ≥4, CPOT ≥3 and BPS ≥6^8 10 33^) was measured, and pain was re-measured within 1 hour (process);
3. Proportion of patient-shifts during which pain was measured and no unacceptable pain scores were observed (outcome);
4. Proportion of patient-shifts during which an unacceptable pain score was re-measured within 1 hour, and the pain score was normalised (outcome).

### Participants

We invited all 82 ICUs that participated in the NICE registration by telephone and email to participate in the trial. ICUs were eligible to participate in the trial if they were willing and able to submit the data items needed to calculate the newly developed pain management indicators monthly in addition to their regular data upload.^30 34^ Furthermore, they had to allocate a multidisciplinary quality improvement team with one person identified as the key contact person for the trial team. Individual patients were excluded if they were delirious, comatose or had a Glasgow coma score <8 because the pain measurement instruments we included were not valid to use in these patient groups.^35^ ICUs started receiving the intervention between January 2016 and November 2017. The medical managers of the participating ICUs signed a consent form to formalise the ICUs’ commitment.^36^

### Intervention: feedback only versus feedback with toolbox

The A&F intervention was informed by Control Theory^22^ and guided by evidence and the latest recommendations for designing A&F.^27 37^ Online supplementary table 2 compares our intervention design with each of Brehaut *et al*’s 15 recent suggestions for optimising A&F. The key component was an online dashboard (screenshot available in online supplementary figure 1) that provided detailed performance information using trend charts, indicator descriptions and patient subgroup analyses. Performance information was updated automatically each time an ICU submitted new data (typically monthly). Indicator performance scores were all calculated over the most recent 3 months.^27^

10.1136/bmjqs-2019-009588.supp2 Supplementary data

10.1136/bmjqs-2019-009588.supp4 Supplementary data

**Figure 1:**
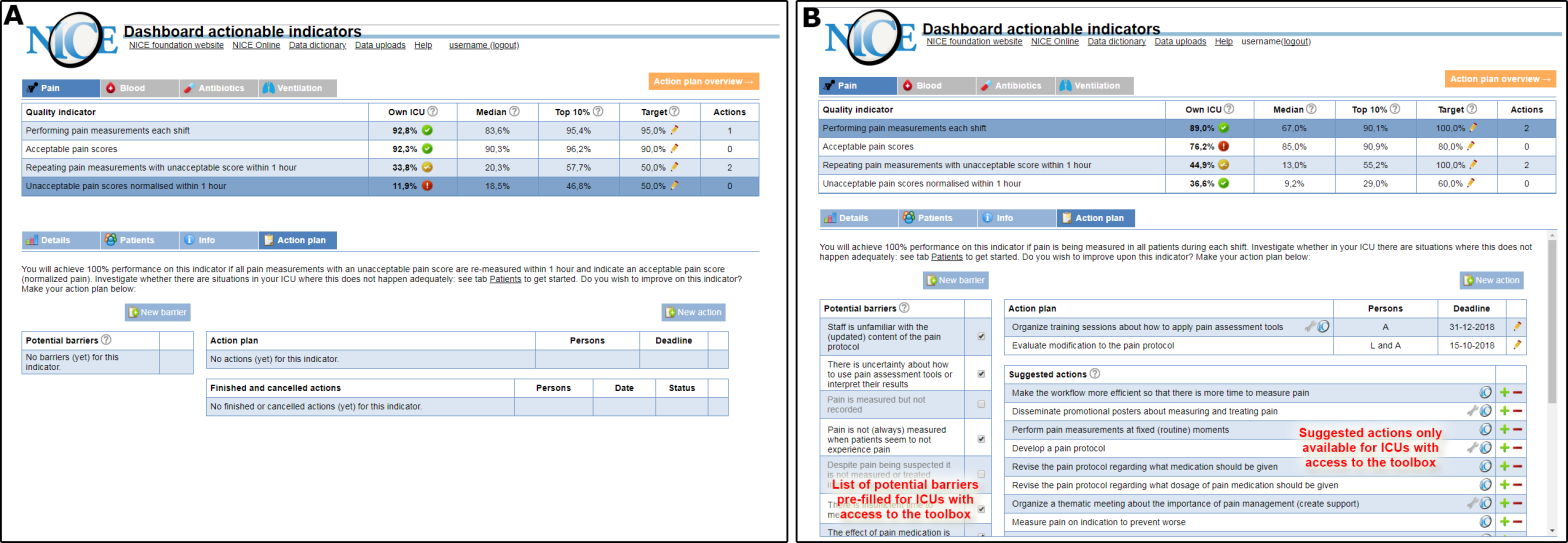
NICE dashboard displayed an overview of pain management performance (upper part) and four types of pages specific to the selected indicator (lower part). the difference between study groups was only in the action plan page. The feedback-only group received an empty structured action plan (A) and could record and update potential barriers and intended actions. The action plan for the feedback-with-toolbox (B) group was supplemented with a pre-filled list of potential barriers and suggested actions (indicated by the NICE icon). Some actions included supporting materials (indicated by a wrench icon) available for download. Users could add suggested actions to their action plan (plus sign) or hide them if they were not relevant (minus sign).

For the *feedback-only* group, the dashboard incorporated an empty action planning template (figure 1A) to facilitate the development and management of structured action plans. The ICUs were asked to list, for each indicator, potential barriers in their care process and actions that they intended to carry out to overcome these barriers. Each action was assigned to one or more people in the ICUs’ quality improvement team, with a set deadline, and additional details could be recorded in free text.

ICUs in the *feedback-with-toolbox* group received the same intervention, but additionally received the action implementation toolbox which was integrated into the action planning page (figure 1B). Consequently, their empty action plan was pre-filled with a list of potential barriers and suggested actions with supporting material to overcome these barriers; the list was the same for all ICUs in the toolbox group, regardless of their performance. Users could select barriers and actions, depending on the relevance and suitability for their local setting.

We developed the action implementation toolbox combining evidence from literature and guidelines and knowledge from ICU experts,^38^ and informed by Flottorp *et al*’s checklist for determinants of practice^39^ and Systems Engineering Initiative for Patient Safety model.^40^ We identified barriers that could potentially lead to poor performance on any of the four pain management indicators. For each barrier, we then listed goal-oriented actions that could be effective in overcoming those barriers to attain higher performance and we collected supporting materials to facilitate their implementation. Together, these barriers, improvement actions and materials formed the action implementation toolbox (see online supplementary table 3 for the full content).

10.1136/bmjqs-2019-009588.supp3 Supplementary data

Each participating ICU received an educational outreach visit at the beginning of the study period to explain the dashboard, the action plan and the toolbox when applicable, to ensure the intervention was implemented as intended. All members of the quality improvement team had access to the online dashboard. After the visit, the NICE researchers called the ICUs every 4–6 weeks to monitor progress and to confirm that their action plans were complete and up-to-date. The ICUs were followed up for 6 months.

### Outcome measures

The primary outcome measure was the proportion of patient-shift observations where pain management was adequate, which we defined as a composite of each of the four indicators (described above). Hence, pain management was considered adequate (0/1) if, for a specific patient during a specific shift (ie, night, day or evening shift), pain was measured at least once (0/1) *and* no unacceptable pain scores were observed (0/1), *OR* unacceptable pain scores were followed up with re-measurement (0/1) *and* normalisation within 1 hour (0/1). Secondary outcome measures were the four indicators individually. Pain was measured with VAS or NRS in patients able to self-report, or with BPS or CPOT in ventilated or sedated patients and defined acceptable or normalised when VAS/NRS <4, CPOT <3 and BPS <6, and unacceptable when VAS/NRS ≥4, CPOT ≥3 and BPS ≥6.^8 10 33^

### Sample size

Based on the 2006 Cochrane review of A&F, we expected the *feedback-only* group to achieve a median absolute improvement of the performance of 4.3% (IQR 0.5%–16%).^17^ We calculated that we would need 24 ICUs with an average cluster size of 600 patient-shift observations in 6 months to have 80% power to detect a significant difference in the performance between the *feedback-only* and *feedback-with-toolbox* group of 10% (with a two-sided unpaired t-test with α=0.05).

### Statistical analysis

The analyses were performed on an intention-to-treat basis. We estimated ORs and 95% CI using mixed-effects logistic regression analysis. We included ‘study group’, ‘time’ (in months) and the interaction term ‘study group×time’ as covariates. With the interaction term, we determined the difference in change during the 6-month follow-up period between the two study groups, that is, the effect of the toolbox. To account for clustering effects for observations within ICUs and repeated measurements in admitted patients, we added random intercepts for each ICU and patient admission.

We performed a regression analysis for the primary and each of the four secondary outcomes, including data until 3 months before the study started (ie, pre-intervention data). We followed this approach to adjust for any differences between the feedback-only and feedback-with-toolbox group prior to the study start.^28 41^ The first month after implementation of the intervention was censored from analysis by coding the ‘study group’ variable as missing during this month to give ICUs time to get acquainted with the intervention and to start using it.

To put the primary and secondary outcomes into context, we analysed for both groups the median number and IQR of total planned and completed actions, and the number and percentage (%) of planned and completed actions per indicator. With Mann-Whitney, we tested whether the numbers differed between the groups. All analyses were performed using R V.3.4.3 (R Foundation for Statistical Computing, Vienna, Austria).

## Results

### Participants

**Figure 2:**
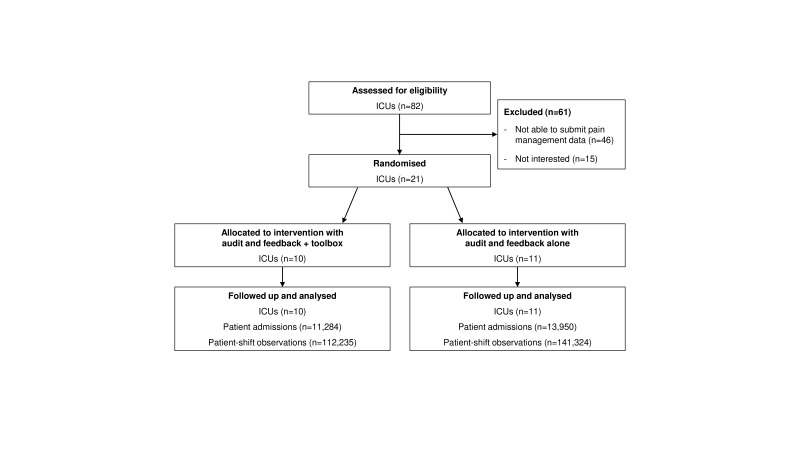
Flow diagram of the progress through the phases of the cluster-randomised controlled trial. ICU, intensive care unit.

From the 82 ICUs that submitted data to the national database, 21 (25.6%) were able to submit the pain management data and provided consent in the timeframe of the study. Eleven ICUs were randomised to the *feedback-only* group and 10 to the *feedback-with-toolbox* group (figure 2). A total number of 25 141 admissions and 253 530 patient-shifts were included in the analyses. Table 1 shows the characteristics of the participating ICUs and patients.

**Table 1:** Characteristics of the participating ICUs and patients; values are median (IQR) unless indicated otherwise

	Feedback only	Feedback with toolbox
ICU-level characteristics		
No included in analysis	11	10
Centre type		
Academic	2	1
Non-academic	9	9
No of beds	16.0 (11.0–24.0)	12.5 (9.0–15.5)
No of FTE intensivists	6.3 (4.9–9.0)	6.1 (5.0–8.6)
No of FTE nurses	64.0 (33.5–86.0)	42.5 (28.4–50.9)
Patient-level characteristics		
No of patient admissions during the trial	13 950	11 284
Admission type		
Surgical (%)	7282 (52.2)	6003 (53.2)
Medical (%)	6639 (47.6)	5269 (46.7)
Unknown (%)	29 (0.2)	12 (0.1)
ICU length of stay in shifts	4.0 (3.0–10.0)	4.0 (3.0–9.0)
Total no of observed patient-shifts	141 324	112 235
Day shift	50 239	39 928
Evening shift	45 957	36 247
Night shift	45 128	36 060

Performance pre-intervention includes 3 months before implementation of the intervention.

FTE, full-time equivalent; ICU, intensive care unit.

### Effect of the feedback intervention

The absolute increase over 6 months in the proportion of patient-shifts with adequate pain management was 14.8% (95% CI 14.0% to 15.5%) in the *feedback-with-toolbox* group and 4.8% (95% CI 4.2% to 5.5%) in the *feedback-only* group. In both groups, we found a significant increase in the proportion of patient-shifts with adequate pain management compared with the pre-intervention period (table 2; OR 1.13 (95% CI 1.06 to 1.22) and OR 1.04 (95% CI 1.00 to 1.09) for the *feedback-with-toolbox* and *feedback-only* group, respectively). The *feedback-with-toolbox* group improved significantly more than the *feedback-only* group (p=0.049). Online supplementary figure 2 shows the performance scores on adequate pain management of the *feedback-only* group and the *feedback-with-toolbox* group over time.

10.1136/bmjqs-2019-009588.supp5 Supplementary data

**Table 2:** Performance scores 3 months before (pre-intervention) and 6 months after implementation of the intervention, and difference in performance between the feedback-only and feedback-with-toolbox group for the primary and secondary outcomes

	Feedback only			Feedback with toolbox		
	Crude pre-intervention performance*	Crude performance after 6 months†	OR (95% CI) at 6 months	Crude pre-intervention performance*	Crude performance after 6 months†	OR (95% CI) at 6 months
Adequate pain management (primary outcome)	69.3% (25 436/36 713)	74.1% (27 128/36 617)	**1.04** **(** **1.00** **to** **1.09)**	59.8% (18 728/31 334)	74.5% (19 789/26 551)	**1.13** **(** **1.06** **to** **1.22)** **‡**
Secondary outcomes						
Performing pain measurements	79.3% (29 115/36 713)	82.7% (30 269/36 617)	**1.04** **(** **1.00** **to** **1.09)**	67.5% (21 138/31 334)	83.1% (22 062/26 551)	**1.24** **(** **1.15** **to** **1.34)** **§**
Re-measuring unacceptable pain within 1 hour	18.6% (804/4318)	23.7% (903/3811)	**1.15** **(** **1.06** **to** **1.25)**	14.3% (390/2735)	24.7% (714/2889)	**1.26** **(** **1.08** **to** **1.47)**
Acceptable pain scores	85.2% (24 797/29 115)	87.4% (26 458/30 269)	0.99 (0.93 to 1.05)	87.1% (18 403/21 138)	86.9% (19 173/22 062)	1.08 (0.97 to 1.20)
Unacceptable pain score normalised within 1 hour	79.5% (639/804)	74.2% (670/903)	0.71 (0.55 to 0.92)	83.3% (325/390)	86.3% (616/714)	1.44 (0.79 to 2.56)

ORs associated with a 6-month increase in time; significant results are shown in bold.

*Pre-intervention performance includes 3 months before implementation of the intervention.

†Performance after 6 months includes months 3 to 6 after implementation of the intervention.

‡Significantly different from the feedback-only group with p=0.049.

§Significantly different from the feedback-only group with p<0.001.

For the secondary outcome measures, ICUs with the toolbox showed a significant improvement on the two process indicators: proportion of patient-shifts with at least one pain measurement and proportion of patient-shifts with unacceptable pain where pain was re-measured within 1 hour (table 2). The absolute increase over 6 months was respectively 15.6% (95% CI 14.9% to 16.3%) and 10.4% (95% CI 8.4% to 12.5%). The improvement on the two outcome indicators was not statistically significant.

The *feedback-only* group also improved significantly on the two process indicators. The absolute increase over 6 months was respectively 3.4% (95% CI 2.8% to 3.9%) and 5.1% (95% CI 3.3% to 6.9%). The outcome indicators did not improve.

ICUs with the toolbox achieved larger improvements on all four indicators compared with the *feedback-only* ICUs, but they only achieved significantly larger improvement on measuring pain each shift (p<0.001).

### Action plans

Throughout the study period, participating ICUs added a total of 234 actions to their action plans (table 3). The *feedback-only* group planned a median of 6 (IQR 5–8.5) actions compared with 9 (7–18.5) in the *feedback-with-toolbox* group (p=0.112), and completed 3 (2.5–5.5) actions compared with 4 (2.5–12.5) in the *feedback-with-toolbox* group (p=0.414). In 87.3% (128 out of 153) of the actions, the *feedback-with-toolbox* group targeted actual practice change compared with 65.4% (53 out of 81) in the *feedback-only* group (p=0.012); the remainder concerned verifications of the underlying feedback data and explorations of potential solutions. In the *feedback-with-toolbox* group, ICUs picked 104 (81.3%) of 128 actions from the toolbox while the other 24 were self-defined. Actions in the group with toolbox addressed a wider variety of practice change than in the group without toolbox. Most actions targeted the process indicators (*feedback-with-toolbox*, 71.2%; *feedback-only*, 80.2%) such as announcements about pain monitoring to increase staff knowledge and skills, individualised feedback if pain has not been measured, and building or adapting reminder systems in the local electronic health record (EHR) to alert when pain should be measured (again). Examples of actions targeted at outcome indicators were revising the pain protocol about pain medication or monitoring and feedback on appropriateness of prescribed pain medication.

**Table 3:** Planned actions and completion rates of planned actions across the indicators in the feedback-only and feedback-with-toolbox group

Pain management indicator	Number (%) of actions planned			Number (%) of actions completed		
	Feedback only (n=81)	Feedback with toolbox (n=153)	P value	Feedback only (n=51)	Feedback with toolbox (n=96)	P value
Performing pain measurements	30 (37.0%)	51 (33.3%)	0.504	19 (37.3%)	37 (38.5%)	0.492
Re-measuring unacceptable pain within 1 hour	35 (43.2%)	58 (37.9%)		22 (43.1%)	31 (32.3%)	
Acceptable pain scores	8 (9.9%)	20 (13.1%)		5 (9.8%)	12 (12.5%)	
Unacceptable pain scores normalised within 1 hour	8 (9.9%)	24 (15.7%)		5 (9.8%)	16 (16.7%)	

## Discussion

The principal finding of this trial was that electronic A&F augmented with an action implementation toolbox resulted in greater improvements in pain management than feedback alone. However, feedback alone was also associated with improvements in pain management over time. Improvement in both groups was mostly due to an increase in measuring pain each shift and on repeating pain measurements within 1 hour after an unacceptable pain score was observed. We found no change in the proportion of patient-shifts with acceptable pain scores, and shifts in which unacceptable pain was normalised within 1 hour. ICUs that received the toolbox achieved larger improvements on all four indicators compared with the other ICUs, but this only reached statistical significance for measuring pain each shift.

The action plans that ICUs had developed during the trial indicated that ICUs with access to the toolbox undertook more actions to improve practice, and targeted a wider variety of practice change, compared with ICUs without toolbox. Furthermore, ICUs without toolbox typically focused on low effort activities to increasing staff awareness and knowledge using existent structures (eg, announcements during regular staff meetings) while ICUs with toolbox more frequently did active efforts to change current workflows (eg, include information about pain status to hand-over moments during shift change). Hence, a toolbox seems a promising approach to initiate more practice change and allow for more rapid iterations of audit and feedback cycles.^42^ Although in our study the contents of the toolbox was equal to all participants with access, there was wide variation between ICUs’ action plans (ie, ICUs picked different actions). This underlines that healthcare organisations have different improvement needs.^43^ It has recently been advocated that we need to go beyond a ‘one size fits all’ approach to implementing A&F.^44^ ICUs were able to tailor the intervention to their local needs by specifying detailed descriptions to the actions they selected. Furthermore, despite the toolbox containing suggested actions for all indicators, improvement was particularly centred around the process indicators (ie, measuring pain each shift and repeating measurements within 1 hour after observing unacceptable pain) and less around the outcome indicators (ie, acceptable and normalised pain scores). This corresponded with ICUs’ action plans, in which by far most (>70%) actions, in both study groups, targeted those process indicators. Improvement on processes is often prioritised over outcomes because they are more actionable.^45^ The actions suggested by the toolbox to improve pain outcomes (eg, increasing nurses’ autonomy to prescribe pain medication) were hardly or never planned or completed by ICUs. It seems that ICUs might have needed support beyond the toolbox, such as additional time, financial or human resources or cultural change, to achieve practice change in their pain management outcomes. A mixed-methods process evaluation carried out alongside this trial investigates the mechanisms through which the toolbox exerted its effects in detail and will be published elsewhere.^27^

Despite the significant improvement on both process indicators, there remained ample room for improvement. For instance, about 76% of patient-shifts where unacceptable pain was observed, pain was not re-measured within 1 hour. Sensitivity analyses showed that this percentage was similar when taking a 2-hour or 3-hour threshold,^32^ which eliminates the hypothesis that the 1 hour may be an unrealistic timeframe in practice. One potential explanation suggested by some of the participating ICUs was that nurses may not have recorded the normalised pain score into the EHR because they considered this administrative task irrelevant or time-consuming. This might have also resulted in an underestimation of the process indicator scores. The lack of improvement in the proportion of shifts with acceptable pain scores may also be explained in part by the fact that pain scores measured in the first shift of patients’ ICU admission are not influenced by pain management in the ICU. Patients from the operating room or from the emergency room may arrive at the ICU with high pain scores. Obviously, this will not be influenced by the quality of pain management provided by the ICU. Another explanation could be that the increase in the number of pain measurements has led to the identification of more shifts in which patients experienced unacceptable pain. The proportion of shifts with unacceptable pain scores that normalised within 1 hour remained constant at around 80%, but this number should be interpreted with caution as it is only based on 24% of shifts in which the pain measurement was actually repeated. This means that the true rate of recorded normalisation was around 19%. We do not know if the rate of normalisation of unacceptable pain scores was comparable in the 76% in which no repeated measurement was performed within 1 hour.

### Strengths and limitations

A strength of this study is that the toolbox provides suggestions for improvement strategies derived from expert opinion, guidelines and scientific literature. We designed our intervention driven by theory^27^ and in accordance with the latest recommendations.^37^ Our head-to-head cluster-RCT, comparing two variations of feedback, contributes to the research field of A&F as traditional intervention versus control RCT no longer advance the science and effectiveness of A&F.^21 25^ However, some limitations warrant discussion. First, baseline pain management performance was lower in the *feedback-with-toolbox* group—despite randomisation. Hence, our finding that ICUs with toolbox improved more than those without toolbox might have been overestimated due to a difference in room for improvement between the two study groups. Due to the limited number of participating ICUs, we were unable to use stratified randomisation based on ICUs’ baseline performance. However, because there remained substantial room for improvement at the study end for both groups, as judged by our clinician coauthors, we argue that the baseline difference has not substantially influenced our estimate of the toolbox’s effect size. Second, our power analysis was based on the composite outcome measure and not on the individual indicators. Especially the indicator on normalising unacceptable pain within 1 hour was a relevant and important indicator when focusing on improving patient care, but with a much lower denominator compared with the other indicators probably underpowered to detect significant effects. Third, we did not find a significant difference in the number of actions between the groups, which can also be attributed to the low number of included ICUs. Finally, measuring pain in patients not able to self-report pain is difficult and might have resulted in underestimated pain scores. However, for these patients, validated tools such as the BPS and CPOT were used.

### Unanswered questions and future research

To explore whether and how A&F interventions in combination with an action implementation toolbox can be applied in routine ICU practice and other clinical settings, we will perform a process evaluation exploring the difference in action planning processes between study groups. Future research should investigate how toolboxes may better facilitate improvement on patient outcomes rather than care processes. It may also explore ways to optimise the design and contents of toolboxes to increase the uptake and usefulness. A starting point could be the creation of a dynamic toolbox where participants may share and use each other’s best practices and experiences, rather than our static list of suggested improvement actions.

## Conclusion

The electronic feedback with toolbox intervention improved the number of shifts where patients received adequate pain management when compared with feedback alone. Improvements focused on process indicators—that is, enhancing the measurement of pain, rather than on outcome indicators—that is, avoiding or addressing unacceptable pain scores. The toolbox seems to be a valuable addition to future A&F interventions.
