# Supplementary material for: Impact of audit and feedback with action implementation toolbox on improving ICU pain management: cluster-randomised controlled trial
Source: BMJ Qual Saf. 2019 Jul 1;28(12):1007–15. doi: 10.1136/bmjqs-2019-009588 (PMC6934240; doi:10.1136/bmjqs-2019-009588)

Supplementary Figure 1A. Screenshot of details page which presents performance scores over time.

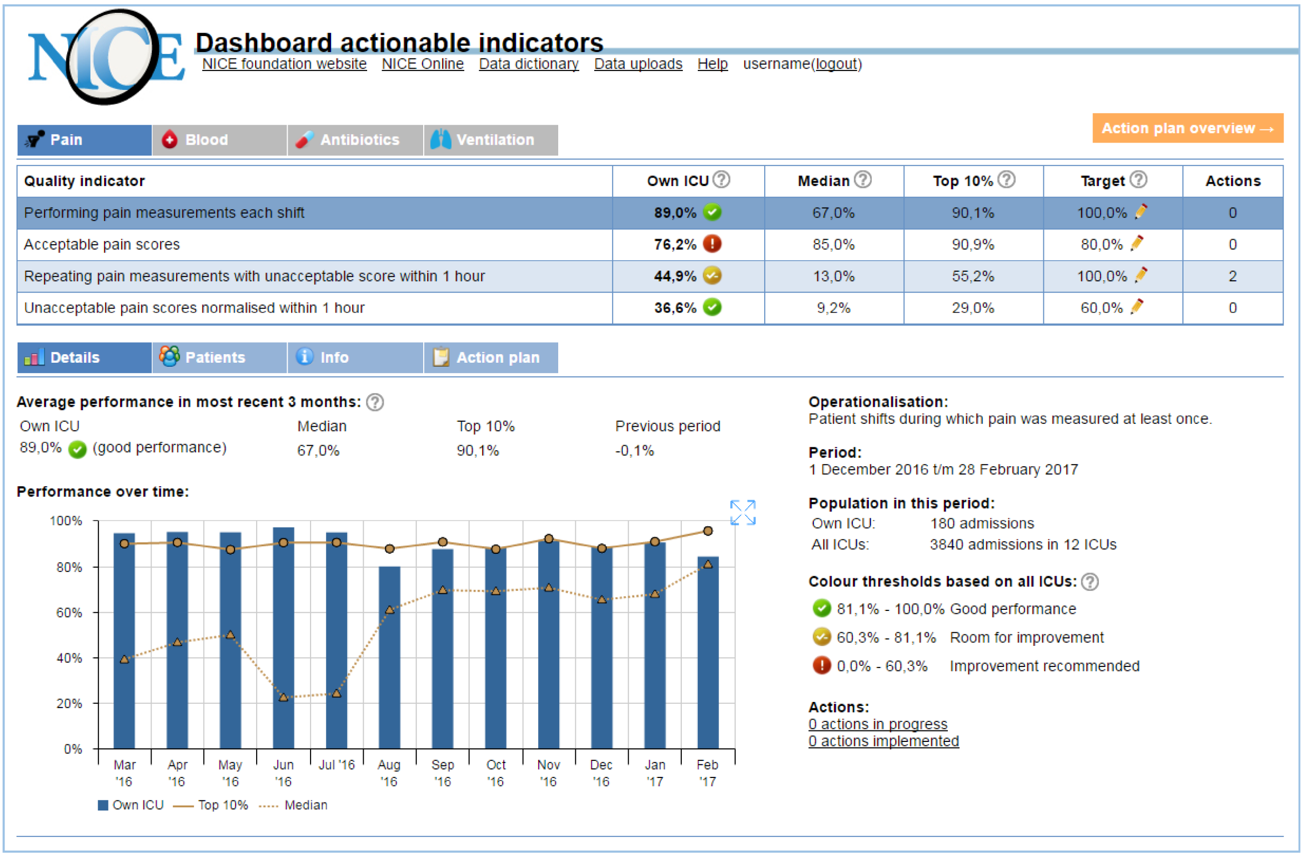

Supplementary Figure 1B. Screenshot of patients page which presents performance scores per subgroup.

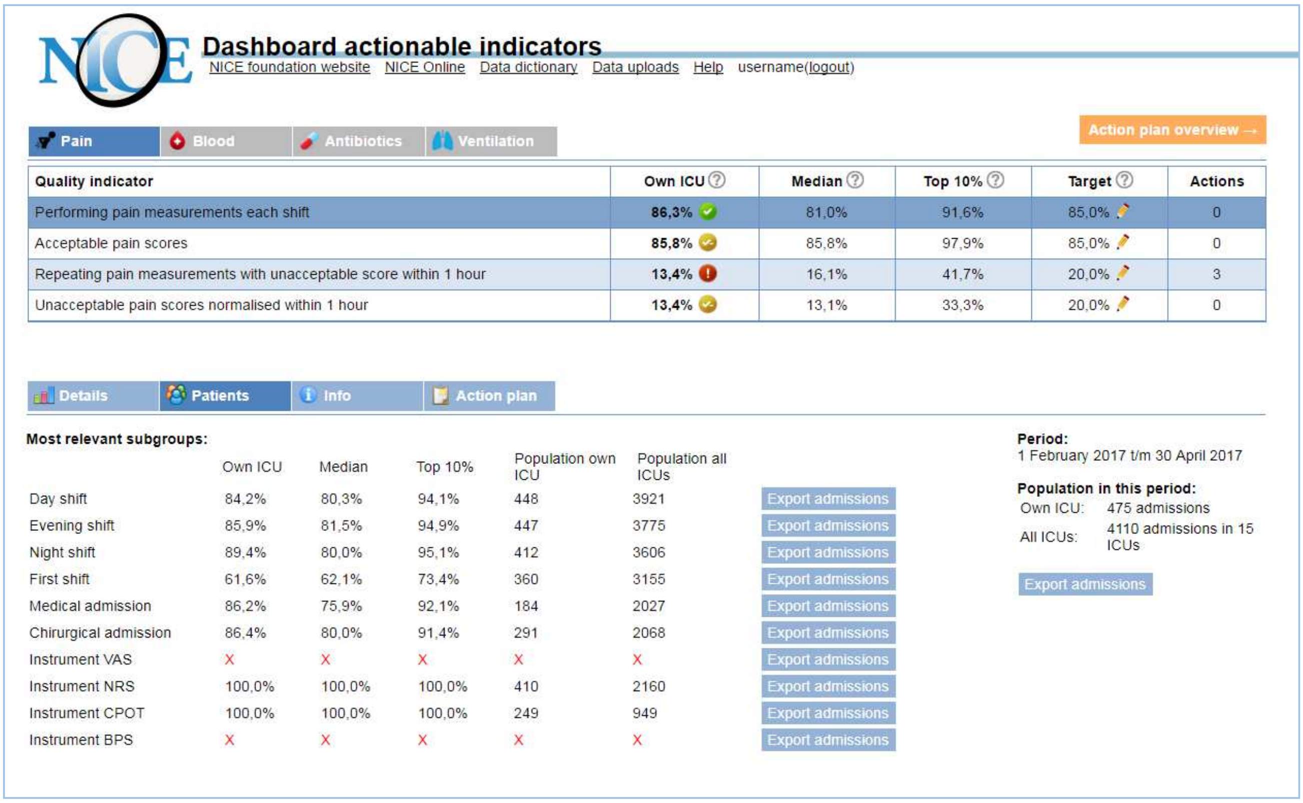

Supplement: Supplementary data [file bmjqs-2019-009588supp001.pdf]
