# Supplementary material for: Impact of audit and feedback with action implementation toolbox on improving ICU pain management: cluster-randomised controlled trial
Source: BMJ Qual Saf. 2019 Jul 1;28(12):1007–15. doi: 10.1136/bmjqs-2019-009588 (PMC6934240; doi:10.1136/bmjqs-2019-009588)
Supplement: Supplementary data [file bmjqs-2019-009588supp003.pdf]

Supplementary Table 1. List of actionable quality indicators to monitor adequate pain management in an ICU.

Indicator	Definition	Indicator type	Numerator	Denominator	Optimal value
1. Performing pain measurements	Percentage of patient-shift observations during which pain was measured at least once.<br><br>*Specified for subgroups: 1) type of admission, 2) type of shift*	Process	Number of patient-shift observations during which pain was measured at least once	Total number of patient-shift observations with or without a pain measurement	100%
2. Re-measuring unacceptable pain within 1 hour#	Percentage of patient-shift observations during which an unacceptable pain score was measured*, and pain was re-measured within one hour<br><br>*Specified for subgroups: 1) type of admission, 2) type of shift*	Process	Number of patient-shift observations during which an unacceptable pain score was measured*, and pain was re-measured within one hour	Total number of patient-shift observations during which an unacceptable pain score was measured*	100%
3. Acceptable pain scores#	Percentage of patient-shift observations during which pain was measured* and no unacceptable pain scores were observed<br><br>*Specified for subgroups: 1) type of admission, 2) type of shift*	Outcome	Number of patient-shift observations during which pain was measured* and no unacceptable pain scores were observed	Total number of patient-shift observations during which pain was measured*	100%
4. Unacceptable pain scores normalized within 1 hour#	Percentage of patient-shift observations during which an unacceptable pain score was measured*, and pain was re-measured within one hour indicating that the pain score was normalized<br><br>*Specified for subgroups: 1) type of admission, 2) type of shift*	Outcome	Number of patient-shift observations during which an unacceptable pain score was measured*, and pain was re-measured within 1 hour indicating that the pain score was normalized	Total number of patient-shift observations during which an unacceptable pain score was measured*	100%

*Only pain scores measured with a standardized pain assessment tool, such as the Visual Analog Scale (VAS), Numerical Rating Scale (NRS), Behavioral Pain Scale (BPS), or Critical-Care Pain Observation Tool (CPOT) are included.
#Unacceptable pain scores were defined as measurements with a VAS or NRS score of ≥4, a CPOT≥3 or a BPS≥6. Acceptable or normalized scores were defined as VAS/NRS<4, CPOT<3 and BPS<6.
