# Supplementary material for: Impact of audit and feedback with action implementation toolbox on improving ICU pain management: cluster-randomised controlled trial
Source: BMJ Qual Saf. 2019 Jul 1;28(12):1007–15. doi: 10.1136/bmjqs-2019-009588 (PMC6934240; doi:10.1136/bmjqs-2019-009588)
Supplement: Supplementary data [file bmjqs-2019-009588supp005.pdf]

Supplementary Table 3. Action implementation toolbox containing possible barriers and subsequent improvement actions on each quality indicator arranged by the determinants of practice from the checklist of Flottorp et al.[1] and Systems Engineering Initiative for Patient Safety (SEIPS) model[2]. The last row on each block e.g. 'Actions 4+5' refer to actions mentioned at another barrier which are also possible improvement strategies for the barrier in question.

A. Barriers relating to the protocol (Work system and structure – Technologies and tools)

**Barrier 1. The pain protocol is inadequate or missing**<br>Relevant to indicator: 1 – 2 – 3 – 4		
**Improvement action**	**Description of improvement action**	**Material**
1. Develop a pain protocol	The pain protocol contains at least information about; the frequency of pain measurements (at least once per shift), the use of valid assessment tools (VAS/NRS for 'communicative' patients, CPOT/BPS for sedated patients), repeat pain measurements in a timely manner (within one hour), pain medication and dosage. An adequate pain protocol can result in higher guideline adherence and better quality of care[3-8].	Standard format protocol
2. Revise the pain protocol regarding what pain medication should be given	If the protocol includes information about appropriate pain medication prescription, it is clear to health professionals how to treat pain effectively[9].	
3. Revise the pain protocol regarding what dosage of pain medication should be given	If the protocol includes adequate information about the dosage of prescribed pain medication, pain can be treated more effectively[9].	
Actions 4+5		

**Barrier 2. 'Measure pain every shift' is not included in the protocol**<br>Relevant to indicator: 1		
**Improvement action**	**Description of improvement action**	**Material**
4. Add the criterion '*Measure pain every shift'* to the protocol	Measuring pain every shift can help to detect and treat pain early. Systemic evaluation is associated with a decrease in pain incidence[5].	

**Barrier 3. 'Repeat pain measurement within one hour in case of an unacceptable score' is not included in the protocol**<br><br>Relevant to indicator: 3 – 4		
**Improvement action**	**Description of improvement action**	**Material**
5. Add the criterion '*Repeat pain measurement within one hour in case of an unacceptable score*' to the protocol	In case pain measurements with an unacceptable score are repeated within one hour to evaluate treatment effect, health professionals can decide on time if therapy should be changed[5, 10].	

**Barrier 4. The pain protocol is (not easily) accessible**<br><br>Relevant to indicator: 1 – 2 – 3 – 4		
**Improvement action**	**Description of improvement action**	**Material**
6. Make the pain protocol available electronically	The pain protocol can be easily accessed when it is available electronically. For example, on a network or online, preferably with a link to it from the electronic health record (EHR) or patient data management system (PDMS)[11].	
7. Develop a flowchart or pocket card with the highlights of the pain protocol	Information of the pain protocol can be clarified by offering a flowchart or pocket card covering essential information from the protocol[11].	Standard format flowchart or pocket card
8. Spread a digital newsletter with details or updates on the pain protocol	Information spread by email is an effective way to introduce people to (updates of) the antibiotic protocol[12].	
9. Provide promotional posters on specific topics relating to pain management	Promotional posters that indicate when and with what instruments pain should be measured, can stimulate familiarity with the protocol and serve as a reminder[13].	Standard format posters
Action 19.		

B. Barriers relating to the individual health care professional (Work system or structure – Person)

**Barrier 5. Health care professionals are not (sufficiently) familiar with the pain protocol**<br>Relevant to indicator: 1 – 2 – 3 – 4		
**Improvement action**	**Description of improvement action**	**Material**
10. Organize an educational meeting on the contents of the pain protocol	Educational sessions help to raise familiarity with the pain protocol, and encourage discussion on the importance of pain measurement[14, 15].<br>It can be helpful to point out a pain coordinator, pain nurse or manager as being responsible for the educational sessions[12].	
Actions 7+8+9+15+18+19+21		

**Barrier 6. Validated pain assessment tools are not always used**<br>Relevant to indicator: 1		
**Improvement action**	**Description of improvement action**	**Material**
11. Measure pain using validated pain assessment tools (VAS, NRS, BPS, CPOT or CIA)	The pain indicators are based on the use of the VAS, NRS, BPS, CPOT, or CIA. It is recommended that one of these assessment tools is being used; VAS or NRS in case of communicative patients, CPOT or BPS in case of sedated patients, CIA in case of non-communicative patients. These assessment tools are validated and proven to measure pain effectively within ICU patients[3, 7, 8].	
Actions 8+9+10+21		

**Barrier 7. Health care professionals do not know (for sure) how to use or interpret pain assessment tools**<br>Relevant to indicator: 1 – 2 – 3 – 4		
**Improvement action**	**Description of improvement action**	**Material**
12. Organize training sessions on the application of pain assessment tools	If training sessions are organized during which pain assessment tools will be explained and difficult situations discussed, this may persuade someone or lower the threshold to use the tools[14].<br>It can be helpful to point out a pain coordinator, pain nurse or manager as being responsible for the educational sessions[12].	Educational PowerPoint presentation

**Barrier 8. Pain is not (always) measured in case health care professionals do not expect a patient to have pain**<br><br>Relevant to indicator: 1 – 3 – 4		
**Improvement action**	**Description of improvement action**	**Material**
13. Measure pain at built-in (routine) moments	When pain is measured in a routine manner the chance to forget pain measurements or miss them due to other reasons is reduced[5].	
Actions 4+8+10+15+19+21		

**Barrier 9. Pain is not measured during shift of admission or discharge**<br><br>Relevant to indicator: 1 – 3		
**Improvement action**	**Description of improvement action**	**Material**
Actions 4+8+10+15+19+21		

**Barrier 10. Despite pain is suspected pain is not measured or treated directly**<br><br>Relevant to indicator: 2 – 3 – 4		
**Improvement action**	**Description of improvement action**	**Material**
14. Measure pain on indication to prevent worse	When pain is measured on indication and not only at regular intervals, it can be treated sooner and worse can be prevented.	
Actions 4+9+10+15+19+21		

**Barrier 11. Effectiveness of pain treatment is not checked sufficiently**<br><br>Relevant to indicator: 2 – 3 – 4		
**Improvement action**	**Description of improvement action**	**Material**
15. Build in alerts in the EHR that remind health care professionals that pain should be measured (again)	Build in decision support in the EHR, reminding of pain measurement every shift and in case of a high pain score after one hour (f.e. with a pop-up), might lead to more adequate decisions in case (high) pain is measured[16, 17]. If the patient is absent from the ICU at the moment the reminder appears (f.e. the patient has left the ICU to get a surgery or MRI), it should be possible to delay the reminder and measure pain later during the shift.	
Actions 4+5+9+10+19		

Barrier 12. Pain scores remain high despite pain treatment<br>Relevant to indicator: 2 – 4		
**Improvement action**	**Description of improvement action**	**Material**
16. Check at random if prescribed pain medication and dosages are correct	Check on the basis of the data in the PDMS or ascertain from random samples whether the correct pain medication and dosage was administered according to the protocol[18].	
17. Improve vigilance on and treatment of underlying factors (such as fear) to prevent pain	It is important to recognize and treat underlying factors, such as fear, because patients experience worse pain if they suffer from underlying factors like fear[10].	
Actions 4+5+9+10+19		

C. Barriers relating to professional interactions (Work system or structure – Organization)

Barrier 13. There is no culture in which measuring pain is considered important<br>Relevant to indicator: 1 – 3		
**Improvement action**	**Description of improvement action**	**Material**
18. Appoint a pain coordinator or team to ensure pain policy	Pain policy and quality improvement initiatives can be ensured by appointing a role model or specific team with pain management as special responsibility[19, 20].	
19. Organize a special meeting on the importance of pain management (create social support)	By organizing an interactive (theme) meeting on the consequences of not measuring pain and prejudices against medication administration, can lead to a better social culture of pain measurement[15]. It can be helpful to point out a pain coordinator, pain nurse or manager as being responsible for the educational sessions[12].	
Actions 8+9+10+21		

Barrier 14. Pain status is not sufficiently communicated at shift change or medical transfer<br>Relevant to indicator: 2 – 3 – 4		
**Improvement action**	**Description of improvement action**	**Material**
20. Ensure pain status is communicated during shift change or medical transfer	When the pain status is handed on sufficiently, by f.e. taking up pain as a fixed part of 'neurologic status' and paying specific attention to high pain scores (VAS/NRS≥4, BPS≥6, CPOT≥3), pain can be treated more appropriately[21]. Furthermore, health professionals know, covering different shifts, when they are expected to measure pain (again).	
Actions 9+10+15+19+21		

D. Barriers relating to incentives and resources (Work system or structure – Technologies and tools)

**Barrier 15. There is not enough time to measure pain**<br><br>**Relevant to indicator: 1 – 3**		
**Improvement action**	**Description of improvement action**	**Material**
21. Provide feedback individually when pain was not being measured	When personnel is fed back individually in case pain was not assessed in every patient during a shift or not repeated within one hour, they may become more conscious of the problem[12].	
22. Increase effectiveness of work process in such way there is more time to measure pain	By facilitating that pain can be measured or registered in the EHR or PDMS more easily and that is less time consuming, pain will be measured more frequently and on time[11, 22].	
Actions 10+13+18+19		

**Barrier 16. Pain is measured but not registered**<br><br>Relevant to indicator: 1 – 2 – 3 – 4		
**Improvement action**	**Description of improvement action**	**Material**
23. Guarantee all measured pain scores are registered in the health record	When pain is measured, assure the score is always registered, even when the patient indicated to have no pain. This way of working guarantees all health professionals to gain a clear understanding of the patient's pain status and to act appropriate to it[23]. It might be of help to oblige pain documentation[12].	
Actions 9+10+11+18+19+21		

**Barrier 17. Pain medication is not prescribed (on time)**<br><br>Relevant to indicator: 2 – 4		
**Improvement action**	**Description of improvement action**	**Material**
24. Take care of standard available prescriptions of pain medication	When prescriptions of pain medication are standard available this can help to start treatment and lower pain earlier[17].	
25. Check for contraindications to pain medications at admission	Existing contraindications can lead to inadequate standard pain medication. By taking up contraindications for pain medication on the checklist (time-out) used at a patient's admission, alternative medication can be discussed early[6, 24].	

26. Increase autonomy or responsibility of nurses to give pain medication	When nurses have more autonomy or responsibility to administer pain medication when needed, treatment of pain can be started sooner, because they are f.e. not dependent of the doctor in attendance. The pain protocol may include the steps nurses can undertake to reduce pain, such as what pain medication nurses can administer when needed, without involvement of the doctor[25].	
Actions 10+19		

**Barrier 18. Responsible doctor is not available on time to prescribe pain medication**<br><br>Relevant to indicator: 2 – 4		
**Improvement action**	**Description of improvement action**	**Material**
Actions 10+24+25+26		

References

1. Flottorp SA, Oxman AD, Krause J, et al. A checklist for identifying determinants of practice: a systematic review and synthesis of frameworks and taxonomies of factors that prevent or enable improvements in healthcare professional practice. *Implement Sci* 2013;8:35 doi: 10.1186/1748-5908-8-35 [published Online First: 2013/03/26].
2. Carayon P, Schoofs Hundt A, Karsh BT, et al. Work system design for patient safety: the SEIPS model. *Qual Saf Health Care* 2006;15 Suppl 1:i50-8 doi: 10.1136/qshc.2005.015842 [published Online First: 2006/12/05].
3. Gelinas C, Fillion L, Puntillo KA, et al. Validation of the critical-care pain observation tool in adult patients. *Am J Crit Care* 2006;15(4):420-7 [published Online First: 2006/07/11].
4. Gelinas C, Harel F, Fillion L, et al. Sensitivity and specificity of the critical-care pain observation tool for the detection of pain in intubated adults after cardiac surgery. *J Pain Symptom Manage* 2009;37(1):58-67 doi: 10.1016/j.jpainsymman.2007.12.022 [published Online First: 2008/07/05].
5. Chanques G, Jaber S, Barbotte E, et al. Impact of systematic evaluation of pain and agitation in an intensive care unit. *Crit Care Med* 2006;34(6):1691-9 doi: 10.1097/01.CCM.0000218416.62457.56 [published Online First: 2006/04/21].
6. Joffe AM, Hallman M, Gelinas C, et al. Evaluation and treatment of pain in critically ill adults. *Seminars in respiratory and critical care medicine* 2013;34(2):189-200 doi: 10.1055/s-0033-1342973 [published Online First: 2013/05/30].
7. Ferreira-Valente MA, Pais-Ribeiro JL, Jensen MP. Validity of four pain intensity rating scales. *Pain* 2011;152(10):2399-404 doi: 10.1016/j.pain.2011.07.005 [published Online First: 2011/08/23].
8. Aissaoui Y, Zeggwagh AA, Zekraoui A, et al. Validation of a behavioral pain scale in critically ill, sedated, and mechanically ventilated patients. *Anesth Analg* 2005;101(5):1470-6 doi: 10.1213/01.ANE.0000182331.68722.FF [published Online First: 2005/10/26].
9. Decosterd I, Hugli O, Tamches E, et al. Oligoanalgesia in the emergency department: short-term beneficial effects of an education program on acute pain. *Ann Emerg Med* 2007;50(4):462-71 doi: 10.1016/j.annemergmed.2007.01.019 [published Online First: 2007/04/21].

10. Hamill-Ruth RJ, Marohn ML. Evaluation of pain in the critically ill patient. *Critical care clinics* 1999;15(1):35-54, v-vi [published Online First: 1999/02/04].
11. Jun J, Kovner CT, Stimpfel AW. Barriers and facilitators of nurses' use of clinical practice guidelines: An integrative review. *Int J Nurs Stud* 2016;60:54-68 doi: 10.1016/j.ijnurstu.2016.03.006 [published Online First: 2016/06/15].
12. Fischer F, Lange K, Klose K, et al. Barriers and Strategies in Guideline Implementation-A Scoping Review. *Healthcare (Basel)* 2016;4(3) doi: 10.3390/healthcare4030036 [published Online First: 2016/07/16].
13. Scales DC, Dainty K, Hales B, et al. A multifaceted intervention for quality improvement in a network of intensive care units: a cluster randomized trial. *JAMA* 2011;305(4):363-72 doi: 10.1001/jama.2010.2000 [published Online First: 2011/01/21].
14. Erdek MA, Pronovost PJ. Improving assessment and treatment of pain in the critically ill., 2004.
15. Lewis CP, Corley DJ, Lake N, et al. Overcoming barriers to effective pain management: the use of professionally directed small group discussions. *Pain management nursing : official journal of the American Society of Pain Management Nurses* 2015;16(2):121-7 doi: 10.1016/j.pmn.2014.05.002 [published Online First: 2014/12/03].
16. Grol R, Grimshaw J. From best evidence to best practice: effective implementation of change in patients' care. *Lancet* 2003;362(9391):1225-30 doi: 10.1016/S0140-6736(03)14546-1 [published Online First: 2003/10/22].
17. van Gulik L, Ahlers SJ, Brkic Z, et al. Improved analgesia after the realisation of a pain management programme in ICU patients after cardiac surgery. *European journal of anaesthesiology* 2010;27(10):900-5 [published Online First: 2010/09/21].
18. Gunningberg L, Poder U, Donaldson N, et al. Medication administration accuracy: using clinical observation and review of patient records to assess safety and guide performance improvement. *J Eval Clin Pract* 2014;20(4):411-6 doi: 10.1111/jep.12150 [published Online First: 2014/05/07].
19. Huis A, Schoonhoven L, Grol R, et al. Impact of a team and leaders-directed strategy to improve nurses' adherence to hand hygiene guidelines: a cluster randomised trial. *Int J Nurs Stud* 2013;50(4):464-74 doi: 10.1016/j.ijnurstu.2012.08.004 [published Online First: 2012/09/04].
20. Hulscher ME, Schouten LM, Grol RP, et al. Determinants of success of quality improvement collaboratives: what does the literature show? *BMJ quality & safety* 2013;22(1):19-31 doi: 10.1136/bmjqs-2011-000651 [published Online First: 2012/08/11].
21. Conroy KM, Elliott D, Burrell AR. Testing the implementation of an electronic process-of-care checklist for use during morning medical rounds in a tertiary intensive care unit: a prospective before-after study. *Ann Intensive Care* 2015;5(1):60 doi: 10.1186/s13613-015-0060-1 [published Online First: 2015/08/05].
22. Cheung A, van Velden FH, Lagerburg V, et al. The organizational and clinical impact of integrating bedside equipment to an information system: a systematic literature review of patient data management systems (PDMS). *International journal of medical informatics* 2015;84(3):155-65 doi: 10.1016/j.ijmedinf.2014.12.002 [published Online First: 2015/01/21].
23. Erdek MA, Pronovost PJ. Improving assessment and treatment of pain in the critically ill. *Int J Qual Health Care* 2004;16(1):59-64 doi: 10.1093/intqhc/mzh010 [published Online First: 2004/03/17].
24. Hamilton-ter Brake AT, Ahlers SJGM, van Gulik L, et al. Intensieve aandacht voor pijnbeleid op de intensivecare-unit; beklijft het effect op de incidentie van onacceptabele pijn na cardiothoracale chirurgie? *PW Wetenschappelijk Platform* 2015;9(A1519):112-16.
25. Hatherley C, Jennings N, Cross R. Time to analgesia and pain score documentation best practice standards for the Emergency Department - A literature review. *Australas Emerg Nurs J* 2016;19(1):26-36 doi: 10.1016/j.aenj.2015.11.001 [published Online First: 2016/01/01].
